# Special Issue “Immune Regulation in Lung Diseases”

**DOI:** 10.3390/ijms27219520

**Published:** 2026-10-24

**Authors:** Chiara Colarusso, Rosalinda Sorrentino

**Affiliations:** 1 Department of Pharmacy (DIFARMA), University of Salerno, Via Giovanni Paolo II, 132, 84084 Fisciano, SA, Italy; 2 Biogem S.c.a r.l., Istituto di Ricerche Genetiche “Gaetano Salvatore”, CaReBios Surl, Contrada Camporeale, 83031 Ariano Irpino, AV, Italy

The lung is continuously exposed to environmental insults, cigarette smoke, air pollutants and pathogens that can trigger immune responses and contribute to the establishment and progression of a wide spectrum of respiratory diseases, including idiopathic pulmonary fibrosis (IPF), chronic obstructive pulmonary disease (COPD), asthma, infectious diseases, and lung cancer [1]. Under physiological conditions, lung homeostasis is maintained through the coordinated activity of structural and immune cells, which interact to ensure effective host defence while limiting excessive inflammation and tissue injury [2]. This delicate balance relies on the dynamic interplay between innate and adaptive immunity, inflammatory mediators, cellular metabolism, and tissue-specific signalling networks. When these mechanisms become dysregulated, immune responses may shift from protective to pathogenic, contributing to persistent inflammation, pathological remodelling, and disease occurrence and progression.

Despite substantial progress in characterizing lung immune responses, the precise mechanisms linking immune-cell activity, metabolic adaptation, inflammatory signalling, and tissue remodelling are still being clarified [3]. The heterogeneity of respiratory diseases and the multifaceted nature of immune regulation underscore the need to investigate the ways in which immune pathways contribute to distinct pathological contexts and how these mechanisms may be exploited for therapeutic benefit [3]. Within this panorama, the present Special Issue brings together four research articles and one review, exploring complementary dimensions of immune regulation in lung disease. Although the contributions address different clinical and experimental settings, they converge on a central question: how do systemic and local immune mechanisms interact with inflammatory signalling, cellular metabolism, and tissue responses to shape disease severity and progression? By addressing this question from different perspectives, the collected contributions illustrate the interconnected nature of pulmonary immune regulation, encompassing systemic biomarkers, innate immune activation, immunomodulatory interventions and metabolic checkpoints, and emerging therapeutic strategies (Figure 1).

In this article collection, the development of an integrative inflammatory-metabolic risk index—Vitamin D Inflammatory Burden Score (VDIBS-Core), which combines vitamin D status, systemic inflammation, and coagulation activation—was described. Building on the knowledge that vitamin D deficiency is common in hospitalized COVID-19 patients and is associated with increased severity [4], the authors considered C-reactive protein (CRP), ferritin, D-dimer and lactate dehydrogenase (LDH) as indices of systemic inflammation, coagulation activation, and ensuing tissue injury. Their results highlighted that the VDIBS-Core showed discriminatory capacity for severe COVID-19 and that the incorporation of vitamin D status into COVID-19 patients’ routine examinations improved the predictive performance compared with inflammatory and coagulation biomarkers alone. Therefore, this composite approach may help support clinical risk stratification and inform decisions regarding treatment intensity and monitoring frequency assignment in hospitalized COVID-19 patients, although further external validation is necessary before widespread clinical implementation. Thus, the findings reported here suggest that VDIBS-Core could represent a potentially useful clinical risk-stratification tool, highlighting that disease severity may reflect the interaction between systemic metabolic status, inflammation, and coagulation rather than the infectious agent alone.

Beyond the identification of circulating biomarkers, understanding the mechanisms driving excessive inflammation is essential for explaining the reason why immune responses become detrimental in severe lung disease. In this scenario, the severity of COVID-19 is often correlated to a status of chronic inflammation, with the inflammasome—a multiprotein intracellular complex that orchestrates inflammatory responses and induces a form of programmed cell death—playing a key role in activating inflammatory interleukins and in affecting mortality in coronavirus infections [5]. In this Special Issue, an observational *post-mortem* study was included, which evaluated the expression of inflammasome-associated molecules in patients who succumbed to COVID-19 during the second wave, investigating genetic polymorphisms in genes associated with inflammatory responses. Furthermore, this research article highlighted that the expression levels of *TLR4*, *NLRP3*, and *IL18* were significantly higher in samples from the second wave than in those from the first wave; moreover, genetic variants in *NFKB1* and *NOX4* were associated with a higher expression of the corresponding proteins. Taken together, these results suggest that lung immune responses may be influenced by both the inflammatory environment and individual genetic variability, particularly in the inflammasome pathway, which—as already reported by others [5]—merits further investigation for its potential as a diagnostic or therapeutic target.

Altogether, the two COVID-19 studies published in this Special Issue address complementary levels of disease-associated immune dysregulation: while VDIBS-Core focuses on the integration of systemic parameters for clinical risk assessment, the inflammasome study explores molecular and genetic features associated with severe inflammation.

Moving from acute infection-associated inflammation to chronic inflammatory and fibrotic conditions, this Special Issue also addresses whether immune modulation can influence the progression of structural lung damage associated with interstitial lung diseases (ILDs). The research article by Kotani et al., 2025 [6] focused on ILD associated with connective tissue disease (CTD-ILD), a severe condition characterized by inflammation and progressive lung fibrosis. Given the limited therapeutic options, human umbilical-cord-derived mesenchymal stem cells (huMSCs) have attracted interest due to their potential anti-inflammatory and anti-fibrotic properties [6]. In this context, the authors questioned whether the therapeutic effects of huMSC activity depended on the administered dose in a bleomycin-induced mouse model, followed by the administration of three different doses of huMSCs. They found that the intermediate dose of 10^4^ cells produced the most pronounced effects in terms of ameliorating fibrosis and collagen deposition, reduction in pro-inflammatory cytokine levels (IL-1β and IL-6), and changes in extracellular matrix remodelling with an ensuing decrease in Tissue Inhibitor of Metalloproteinases 1 (TIMP-1) and increase in Matrix Metalloproteinase 9 (MMP-9). Interestingly, the treatment with huMSCs affected macrophage polarization and markedly attenuated the pro-inflammatory M1-associated phenotype. Thus, these results connect immune modulation with changes in the fibrotic lung environment, suggesting that the therapeutic effects of huMSCs may involve coordinated alterations in inflammatory responses and extracellular matrix remodelling. Furthermore, the authors also highlighted the importance of dose optimization to balance efficacy and safety [6]; nevertheless, these findings are still preclinical and further investigation is required before their relevance to the treatment of ILD patients can be established.

While the huMSC study investigates a potential therapeutic strategy to modulate inflammation and fibrosis, one of the contributions of this Collection examines a more fundamental question: in which ways does cellular metabolism support immune-cell function during acute pulmonary infection? In this research article, the role of glucose transporter 1 (GLUT1) in myeloid immune response during *Streptococcus pneumoniae* infection was investigated. To this end, experiments were performed on monocytes and neutrophils from patients with community-acquired pneumonia, and mice with myeloid-cell-specific GLUT1 deficiency. It was shown that *GLUT1* mRNA expression was increased in monocytes and neutrophils obtained from pneumonia patients compared with controls, and that ex vivo stimulation increased GLUT1 protein expression. Surprisingly, they found that myeloid-specific GLUT1 deficiency in mice did not substantially alter the responses to pneumococcal pneumonia, including bacterial burden, lung pathology, and cytokine responses. On the one hand, these data suggested that GLUT1 may contribute to a myeloid-cell-mediated response during pneumococcal pneumonia; on the other hand, they highlighted that immune regulation in the lung depends not only on metabolic activation but also on the flexibility of cellular metabolic programmes.

Importantly, these findings distinguish the association between metabolic activation and immune responses from the demonstration of a direct functional requirement for a specific metabolic transporter. Although increased GLUT1 expression accompanied myeloid-cell activation in patients, its deficiency in the experimental model did not substantially compromise the measured responses to infection. This observation suggests that the relationship between cellular metabolism and immune function may be more complex than the modulation of a single glucose transporter, potentially involving compensatory mechanisms or alternative metabolic pathways. Thus, the study highlights the importance of investigating metabolic flexibility when seeking to understand the ways in which immune cells maintain their function under pathological conditions.

The role of metabolism in shaping immune-cell function provides a natural link between the experimental findings on GLUT1 and the broader mechanisms discussed in the unique review in this Special Issue, which investigated how metabolic programmes in lung dendritic cells (DCs)—key sentinels in the lung mucosa—influence direct tolerance and lymphoid neogenesis in chronic lung inflammation. It was highlighted that lung inflammatory stimuli can activate metabolic pathways involving Hypoxia-Inducible Factor 1-Alpha (HIF-1α) and mammalian Target of Rapamycin (mTOR), increasing glycolytic activity and supporting DC maturation, cytokine production, antigen presentation, and migration. Particularly, specific metabolic checkpoint programmes—such as AMP-activated protein kinase (AMPK), oxidative metabolism, and lipid metabolism—can favour more tolerogenic dendritic cell states, which, in turn, may promote regulatory T-cell responses and help limit excessive inflammation. Moreover, conventional DC type 1 (cDC1) and cDC2 subsets interact with stromal cells and lymphocytes and contribute to the formation of tertiary lymphoid structures, able to support persistent local immune responses further contributing to chronic inflammation and tissue remodelling associated with several pulmonary diseases, including asthma, COPD, pulmonary hypertension, and fibrotic interstitial lung diseases.

Taken together, the research articles collected in this Special Issue investigated different levels of a common biological problem: the ways in which the immune system coordinates metabolism, inflammation, and tissue repair to maintain pulmonary homeostasis and to avoid the establishment and/or progression of lung diseases.

A central message emerging from this Collection is that lung immune regulation cannot be understood by examining individual biomarkers, cell populations, or signalling pathways in an independent mode, due to all these processes being interconnected. When immune responses fail to resolve appropriately, persistent inflammation can contribute to tissue injury and pathological repair. Understanding these interconnected mechanisms may therefore reveal therapeutic opportunities to restore immune homeostasis, preserve protective immunity, and limit progressive lung damage.

In conclusion, the contributions collected in this Special Issue reinforce the importance of lung immune regulation as a common, yet context-dependent, determinant in health and disease. By integrating insights from systemic inflammation, innate immunity, immunomodulation, and cellular metabolism, this Collection provides a foundation for future research aimed at translating a deeper understanding of lung immune mechanisms into improved diagnostic and therapeutic strategies.

## Figures and Tables

**Figure 1:**
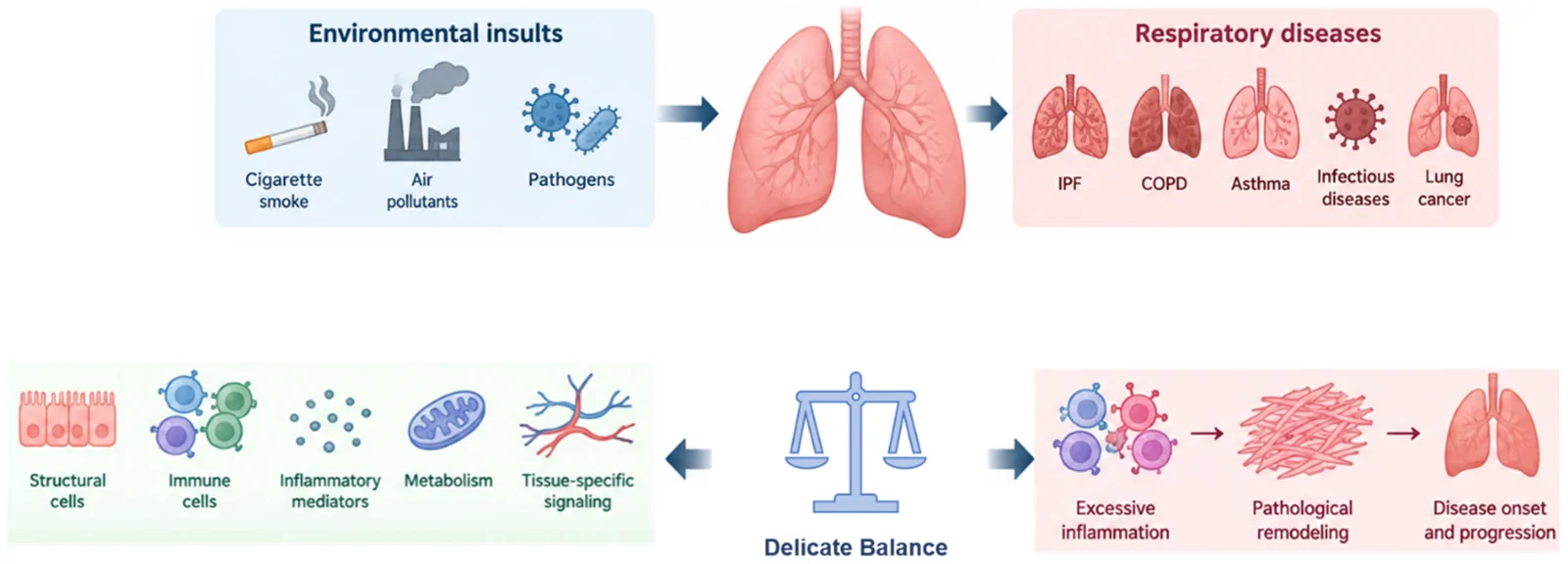
Schematic representation of immune regulation in lung diseases. Several insults can disrupt lung immune homeostasis, leading to inflammation, tissue remodelling, and respiratory diseases. This figure was generated with the assistance ofChatGPT (OpenAI), GPT-6, and manually refined by the authors to ensure scientific accuracy.

## Data Availability

No new data were created or analyzed in this study. Data sharing is not applicable to this article.

## References

[1] Schraufnagel D.E., Balmes J.R., Cowl C.T., De Matteis S., Jung S.H., Mortimer K., Perez-Padilla R., Rice M.B., Riojas-Rodriguez H., Sood A. (2019). Air Pollution and Noncommunicable Diseases: A Review by the Forum of International Respiratory Societies’ Environmental Committee, Part 1: The Damaging Effects of Air Pollution. Chest.

[2] Lloyd C.M., Marsland B.J. (2017). Lung homeostasis: Influence of age, microbes and the environment. Nat. Rev. Immunol..

[3] Yuan Z., Alhaskawi A., Tang Y., Ruan B. (2026). Recent understanding of immunometabolic remodeling in pulmonary macrophages: Homeostasis, chronic respiratory diseases, and therapeutic targeting. Front. Pharmacol..

[4] D’Ecclesiis O., Gavioli C., Martinoli C., Raimondi S., Chiocca S., Miccolo C., Bossi P., Cortinovis D., Chiaradonna F., Palorini R. (2022). Vitamin D and SARS-CoV2 infection, severity and mortality: A systematic review and meta-analysis. PLoS ONE.

[5] Colarusso C., Terlizzi M., Maglio A., Molino A., Candia C., Vitale C., Hansbro P.M., Vatrella A., Pinto A., Sorrentino R. (2022). Activation of the AIM2 Receptor in Circulating Cells of Post-COVID-19 Patients With Signs of Lung Fibrosis Is Associated With the Release of IL-1α, IFN-α and TGF-β. Front. Immunol..

[6] Kotani T., Saito T., Masutani R., Uemura S., Matsuda S., Suzuka T., Ikemoto M., Takeuchi T. (2025). Evaluating the Dose-Dependent Effects of Human Umbilical Cord-Derived Mesenchymal Stem Cells in a Preclinical Model of Interstitial Lung Disease. Int. J. Mol. Sci..

